# Comparing the Hospital Frailty Risk Score and the Clinical Frailty Scale Among Older Adults With Chronic Obstructive Pulmonary Disease Exacerbation

**DOI:** 10.1001/jamanetworkopen.2022.53692

**Published:** 2023-02-02

**Authors:** Melanie Chin, Tetyana Kendzerska, Jiro Inoue, Michael Aw, Linda Mardiros, Christopher Pease, Melissa K. Andrew, Smita Pakhale, Alan J. Forster, Sunita Mulpuru

**Affiliations:** 1 Division of Respirology, Department of Medicine, University of Ottawa, Ottawa, Ontario, Canada; 2 The Ottawa Hospital Research Institute, Clinical Epidemiology Program, Ottawa, Ontario, Canada; 3 The Ottawa Hospital, Ottawa, Ontario, Canada; 4 Faculty of Medicine, University of Ottawa, Ottawa, Ontario, Canada; 5 Department of Medicine (Geriatrics), Dalhousie University, Halifax, Nova Scotia, Canada

## Abstract

**Importance:**

Frailty is associated with severe morbidity and mortality among people with chronic obstructive pulmonary disease (COPD). Interventions such as pulmonary rehabilitation can treat and reverse frailty, yet frailty is not routinely measured in pulmonary clinical practice. It is unclear how population-based administrative data tools to screen for frailty compare with standard bedside assessments in this population.

**Objective:**

To determine the agreement between the Hospital Frailty Risk Score (HFRS) and the Clinical Frailty Scale (CFS) among hospitalized individuals with COPD and to determine the sensitivity and specificity of the HFRS (vs CFS) to detect frailty.

**Design, Setting, and Participants:**

A cross-sectional study was conducted among hospitalized patients with COPD exacerbation. The study was conducted in the respiratory ward of a single tertiary care academic hospital (The Ottawa Hospital, Ottawa, Ontario, Canada). Participants included consenting adult inpatients who were admitted with a diagnosis of acute COPD exacerbation from December 2016 to June 2019 and who used a clinical care pathway for COPD. There were no specific exclusion criteria. Data analysis was performed in March 2022.

**Exposure:**

Degree of frailty measured by the CFS.

**Main Outcomes and Measures:**

The HFRS was calculated using hospital administrative data. Primary outcomes were the sensitivity and specificity of the HFRS to detect frail and nonfrail individuals according to CFS assessments of frailty, and the secondary outcome was the optimal probability threshold of the HFRS to discriminate frail and nonfrail individuals.

**Results:**

Among 99 patients with COPD exacerbation (mean [SD] age, 70.6 [9.5] years; 56 women [57%]), 14 (14%) were not frail, 33 (33%) were vulnerable, 18 (18%) were mildly frail, and 34 (34%) were moderately to severely frail by the CFS. The HFRS (vs CFS) had a sensitivity of 27% and specificity of 93% to detect frail vs nonfrail individuals. The optimal probability threshold for the HFRS was 1.4 points or higher. The corresponding sensitivity to detect frailty was 69%, and the specificity was 57%.

**Conclusions and Relevance:**

In this cross-sectional study, using the population-based HFRS to screen for frailty yielded poor detection of frailty among hospitalized patients with COPD compared with the bedside CFS. These findings suggest that use of the HFRS in this population may result in important missed opportunities to identify and provide early intervention for frailty, such as pulmonary rehabilitation.

## Key Points

**Question:**

When screening for frailty among hospitalized individuals with chronic obstructive pulmonary disease (COPD), how do frailty measurements based on administrative data (ie, the Hospital Frailty Risk Score) perform against real-time bedside assessments of frailty (ie, the Clinical Frailty Scale)?

**Findings:**

In this clinical practice, cross-sectional study of 99 hospitalized patients with COPD exacerbation, the Hospital Frailty Risk Score had poor sensitivity (27%) to detect frailty compared with the Clinical Frailty Scale assessment.

**Meaning:**

These findings suggest that use of the Hospital Frailty Risk Score, which relies on administrative health data to identify frailty among patients with COPD, may misclassify frail patients as nonfrail, thus missing important opportunities for early identification and intervention to improve frailty.

## Teaser

This cross-sectional study examines the agreement between the Hospital Frailty Risk Score and the Clinical Frailty Scale among hospitalized individuals with chronic obstructive pulmonary disease (COPD) and examines the sensitivity and specificity of the scales to detect frailty.

## Introduction

Frailty is a multidimensional syndrome characterized by progressive physiologic decline that leads to increased vulnerability to health stressors and acute illness.^1,2^ It can be conceptualized and objectively measured on a continuum from well to severely frail. Progressive degrees of frailty are associated with increased risks of death, need for institutional care, increased health care costs, and reduced quality of life.^2,3,4,5^ As individuals with chronic disease live longer, assessing and improving frailty and its consequences among patients with chronic diseases is arguably the most pressing issue facing health systems globally.^6,7,8^

Among individuals with chronic obstructive pulmonary disease (COPD), 19% are frail and 56% are prefrail.^9^ Frailty is associated with frequent hospitalization, longer hospital stays, increased costs, increased mortality, and poor quality of life for these individuals.^9,10,11,12,13,14,15^ Clinical interventions such as comprehensive pulmonary rehabilitation, if initiated early in the trajectory of frailty, have the potential to treat, improve, and even reverse the degree of frailty among individuals with COPD.^14^ For this reason, identifying the degree of frailty can be used as a clinical assessment tool to risk-stratify patients who are at increased risk of further functional decline and who may benefit from nonpharmacological interventions, such as pulmonary rehabilitation, and care planning (ie, goals of care and advanced directives).^14,15,16,17^

In the clinical setting, the comprehensive geriatric assessment is the reference standard for identifying and measuring frailty.^18^ However, applying the comprehensive geriatric assessment at the bedside is time consuming and not easily performed by non–geriatric medicine specialists.^19^ Multiple validated tools to operationalize frailty assessments at the bedside exist that identify degrees of frailty on the basis of gradations of functional impairment or by specific clinical features.^2,20,21^ One such tool is the Clinical Frailty Scale (CFS), which is a validated and widely used bedside frailty assessment instrument that assesses cognition, comorbidity, and function to produce a frailty score on a 9-point scale, ranging from very fit to terminally ill.^2,22^ There are also population-based administrative data indices that use demographic, comorbidity, and prior hospitalization data to determine the presence or risk of frailty.^23^ Although administrative data measures come with advantages of large numbers and routine applicability without additional clinical resource use, they come with potential limitations in terms of the relevance and availability of contributing data points and the lack of contextual clinical judgment.^24^ To understand how administrative data–based frailty assessment tools may be used to identify patients with chronic disease at risk of prefrailty and frailty, a direct comparison between administrative data–based and bedside frailty assessment tools is needed.

In this study, we investigated the degree of agreement between a bedside frailty assessment instrument, the CFS, and an administrative data–based frailty assessment instrument, the Hospital Frailty Risk Score (HFRS), among hospitalized patients with COPD exacerbation. We determined the performance test characteristics of the HFRS (vs the CFS) and then assessed the discrimination of the HFRS in identifying individuals who were deemed vulnerable (ie, prefrail) by the CFS. We hypothesized that the HFRS would not accurately capture individuals who were vulnerable (ie, prefrail) on the basis of the bedside CFS assessment among patients with COPD.

## Methods

### Study Design, Setting, and Participants

**Figure 1.**
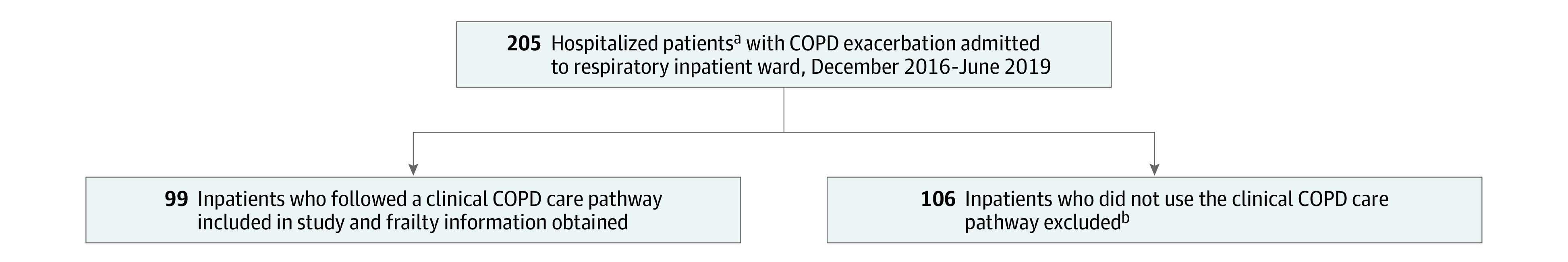
Study Flow Diagram Describing Proportion of Hospitalized Patients With Chronic Obstructive Pulmonary Disease (COPD) Included in the Cross-sectional Analysis ^a^We captured unique hospital admissions during the study time period. Readmissions from the same patient were not captured. ^b^The mean (SD) age of this group was 71.8 (9.9) years; 57 patients (53.8%) were women, 49 patients (46.2%) were men, and the median (IQR) Elixhauser Comorbidity Index score was 3 (3-8).

We conducted a cross-sectional observational study in the respiratory ward of a large, tertiary care, academic center, The Ottawa Hospital, in Ottawa, Ontario, Canada, from December 2016 to June 2019. We studied individuals hospitalized with COPD exacerbation who used a clinical COPD care pathway to guide assessment, treatment, and discharge planning, on a consecutive and voluntary basis. Individual hospital records were reviewed for demographic, comorbidity, and functional status data. There were no specific exclusion criteria. Patients provided written informed consent. The study received local institutional research ethics board approval and is reported in accordance with the Strengthening the Reporting of Observational Studies in Epidemiology (STROBE) reporting guideline. Figure 1 describes the study population.

### Variables and Exposures

We collected demographic (age and sex) and medical comorbidity variables from the records of study participants. Frailty was measured using 2 instruments, the CFS and the HFRS.^2,23^ Two respiratory physicians (M.C. and S.M.) retrospectively assessed each patient’s degree of frailty using the CFS, according to the functional capacity information available in each clinical record. The assessment was based on the patient’s self-reported baseline functional capacity at least 2 weeks before hospitalization (ability to perform activities of daily living and instrumental activities of daily living), which is detailed in the admission notes for patients who used the clinical COPD pathway. The CFS is a validated instrument that captures progressive degrees of frailty according to an individual’s ability to perform basic and instrumental activities of daily living. The CFS is widely applied and validated in many clinical and community settings and has also been validated for use retrospectively in acute care.^22,25^ The CFS assigns frailty groups from 1 (very fit with robust health) to 9 (terminally ill) (eFigure in Supplement 1). For this analysis, we combined well and managing well, and moderately frail and severely frail because the degree of functional performance in each of the combined groups is similar.

HFRS scores were calculated using hospital administrative data according to the methods adapted from Gilbert et al.^23^ Administrative data were obtained from The Ottawa Hospital Data Warehouse, a relational database containing clinical information from the hospital’s operational systems. Comorbidity data in this database are dependent on *International Statistical Classification of Diseases and Related Health Problems, Tenth Revision (ICD-10)*–coded health diagnoses in each patient record. The HFRS is used to identify hospitalized patients with frailty on the basis of prior clinical diagnoses coded with the *ICD-10* system.^23^ A numerical score is determined by the number of relevant *ICD-10* codes from an individual’s prior hospitalization. The risk of frailty is categorized as low (<5 points), intermediate (5-15 points), or high (>15 points).^23^

### Statistical Analysis

Data analysis was performed in March 2022 using SAS statistical software version 9.4 (SAS Institute). Descriptive statistics were used to characterize the cohort’s demographics, comorbidity, and categories of frailty as measured by CFS. We presented individuals by their CFS groups, because the CFS categorizations are a representation of an individual’s empirical and clinically assessed functional status. Means and SDs were compared with analysis of variance testing, medians and IQRs were compared with the Kruskal-Wallis test, and proportions were compared with χ^2^ testing. Two-sided *P* < .05 was considered significant.

We used cross-tabulation between CFS and HFRS frailty groups to describe the degree of agreement between instruments. We calculated sensitivity and specificity of the HFRS to detect frail vs not frail individuals according to the CFS groups. We used the CFS assessments as a criterion standard for this analysis, given its widespread validation in many clinical settings.^22^ For this analysis, the frail group included the vulnerable, mildly frail, and moderately or severely frail categories on the CFS instrument, and the intermediate-risk and high-risk categories on the HFRS instrument. Not frail included the well and managing well categories on the CFS instrument and the low-risk group on the HFRS instrument.

We used receiver operating curve (ROC) analyses and determined the optimal probability threshold by maximizing the Youden J index to identify the HFRS numerical value, which discriminates patients as frail vs not frail according to the CFS measurements.^26^ We did not further stratify the ROC curves analyses by sex given the limitation of our sample size, and additional covariates were not included in the ROC analyses.

## Results

Among 205 unique hospitalized patients admitted with COPD exacerbation during the study period, 99 individuals (48.3%) (mean [SD] age, 70.6 [9.5] years; 56 women [57%]) used a COPD clinical care pathway and were included in our cross-sectional frailty analysis (Figure 1). Table 1 describes the cohort’s demographics and comorbidities. In the included vs excluded cohorts, the female proportion was 56.6% vs 53.8%, and the mean age range was 66.9 to 72.7 years vs 71.8 years. The mean (SD) age increased as the degree of frailty increased (66.9 [9.9] years among those who were well or managing well vs 72.2 [10.1] years among those who were moderately to severely frail). There was a higher proportion of women in each group as the degree of frailty increased (4 women [28.6%] among those who were well or managing well vs 25 women [73.5%] among those who were moderately to severely frail). Heart disease was highly prevalent in this cohort (40 patients [40.4%]). The median (IQR) Elixhauser Comorbidity Index score was higher among individuals who were moderately to severely frail vs those who not frail (well or managing well) (7 [3-8] vs 3 [3-8]). In cross-tabulation of HFRS measurements against CFS frailty groups, median (IQR) HFRS scores remained in the low-risk range (<5 points) across all CFS groups. Using the CFS, 14 of 99 patients (14%) were assessed as well or managing well, 33 (33%) were assessed as vulnerable, 18 (18%) were mildly frail, and 34 (34%) were moderately or severely frail. In total, 85 of 99 patients (86%) were categorized as frail using the CFS in this cohort.

**Table 1.** Baseline Characteristics and Distribution of Frailty by Clinical Frailty Scale Among Hospitalized Individuals With Chronic Obstructive Pulmonary Disease

Characteristic	Patients, No. (%)				*P* value
	Well or managing well (n = 14)	Vulnerable (n = 33)	Mildly frail (n = 18)	Moderately or severely frail (n = 34)	
Age, mean (SD), y	66.9 (9.9)	70.4 (9.2)	70.5 (8.4)	72.2 (10.1)	.37
Sex					
Female	4 (28.6)	16 (48.5)	11 (61.1)	25 (73.5)	.02
Male	10 (71.4)	17 (51.5)	7 (38.9)	9 (26.5)	
Comorbidities					
Heart disease	6 (42.9)	10 (30.3)	5 (27.8)	19 (55.9)	.11
Kidney disease	0	1 (3.0)	0	0	.57
Cancer	0	3 (9.1)	1 (5.6)	3 (8.8)	.68
Depression	0	1 (3.0)	0	1 (2.9)	.81
Weight loss	0	0	1 (5.6)	4 (11.8)	.13
Elixhauser Comorbidity Index score, median (IQR)	3 (3-8)	3 (3-8)	3 (3-8)	7 (3-8)	.83
Hospital Frailty Risk Score, median (IQR)^a^	1 (0-4)	2 (0-4)	2 (0-4)	4 (2-7)	.03

^a^ Hospital Frailty Risk Score groups are low (<5 points), intermediate (5-15 points), and high (>15 points).

Table 2 demonstrates the cross-tabulation of individuals within CFS frailty groups, as they corresponded to the HFRS risk groups (low, intermediate, and high risk). Of 99 individuals in the cohort, 75 (76%), were categorized as low risk (<5 points) for frailty by the HFRS. The HFRS identified 13 of 14 patients (93%) as low risk for frailty, who were nonfrail according to the CFS. However, 20 of 34 patients (59%) who were moderately or severely frail by the CFS were also classified in the low-risk frailty group. Among individuals who were vulnerable on the CFS, only 5 of 33 (15%) were classified in the intermediate-risk frailty group, and among those who were mildly frail on the CFS, only 4 of 18 (22%) were classified in the intermediate-risk frailty group.

**Table 2.** Cross-Tabulation of CFS Groups and HFRS Groups

CFS groups	HFRS groups, patients, No. (%)		
	Low risk (<5 points)	Intermediate risk (5-15 points)	High risk (>15 points)
Well or managing well (n = 14)	13 (92.9)	0	1 (7.1)
Vulnerable (n = 33)	28 (84.8)	5 (15.2)	0
Mildly frail (n = 18)	14 (77.8)	4 (22.2)	0
Moderately or severely frail (n = 34)	20 (58.8)	12 (35.3)	2 (5.9)

Abbreviations: CFS, Clinical Frailty Scale; HFRS, Hospital Frailty Risk Score.

We calculated the sensitivity and specificity of the HFRS to detect frail and nonfrail individuals according to the CFS scoring as a criterion standard measurement (eTable in Supplement 1). The HFRS was 27% sensitive and 93% specific in detecting frail individuals on the basis of the CFS (vulnerable, mildly frail, and moderately or severely frail categories).

**Figure 2.**
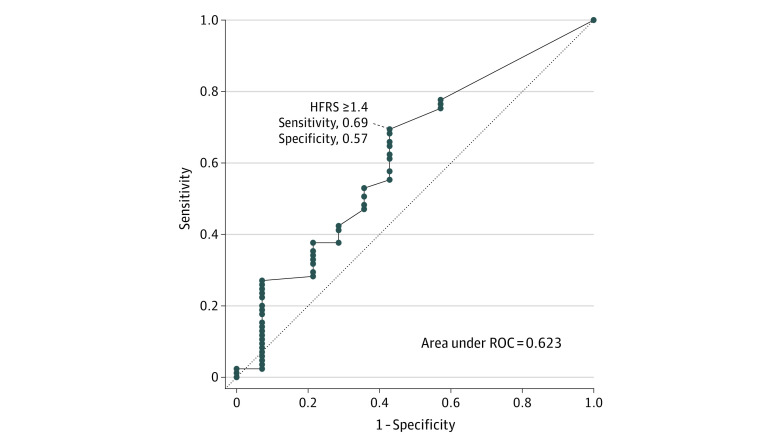
Receiver Operating Curve (ROC) Analysis With Optimal Probability Threshold for Hospital Frailty Risk Score (HFRS)

Figure 2 shows the ROC curve for the HFRS to discriminate frail and nonfrail individuals. The area under the ROC curve is 0.623, suggesting a poor ability for the HFRS to detect frailty scored by CFS. The optimal probability threshold of the HFRS was determined to be a score of 1.4 points or higher. HFRS values greater than or equal to 1.4 had a sensitivity of 69% and specificity of 57% to detect frailty as classified by the CFS. Using the threshold HFRS score of 1 point, we performed the cross-tabulation between CFS and HFRS frailty groups (Table 3). Using this optimal HFRS score, 24 of 33 patients (73%) were identified as moderate or high risk for frailty if they were vulnerable on the CFS instrument. The threshold value of 1 point on the HFRS improved the ability of the HFRS instrument to detect frail individuals, on the basis of CFS categorization. However, 9 of 33 patients (27%) were still found to be low risk for frailty even when they were vulnerable on the CFS instrument.

**Table 3.** Cross-Tabulation of the HRFS and CFS Frailty Groups, Using a HFRS Threshold Score of 1 Point

CFS groups	HFRS groups, patients, No. (%)	
	Low risk (≤1 points)	Moderate or high risk (>1 point)
Well or managing well (n = 14)	6 (42.9)	8 (57.1)
Vulnerable (n = 33)	9 (27.3)	24 (72.7)
Mildly frail (n = 18)	6 (33.3)	12 (66.7)
Moderately or severely frail (n = 34)	6 (17.6)	28 (82.4)

Abbreviations: CFS, Clinical Frailty Scale; HFRS, Hospital Frailty Risk Score.

## Discussion

In a cross-sectional cohort of hospitalized patients with exacerbations of COPD, frailty was very common (86%) according to the degrees of functional impairment captured by the CFS. However, the majority (73%) of patients living with frailty (by CFS measurement) were deemed low risk for frailty when measured by the HFRS. Our results demonstrate disagreement between the HFRS and CFS in categorizing patients as frail. The HFRS demonstrated poor sensitivity (27%) to detect frailty but showed higher specificity (93%). When using the optimal probability threshold of the HFRS (≥1.4 points), the performance was improved, with a higher sensitivity of 69% but lower specificity of 57% to detect frailty on the basis of the CFS classification. Despite this conservative HFRS threshold, 27% of patients who were vulnerable by CFS (which is a prefrail state) were still not captured as being at risk for frailty by the HFRS. Moreover, 33% of those who were already mildly frail on the basis of functional status (CFS) were categorized by HFRS as low risk for frailty.

Although there is a discrepancy between the CFS and HFRS assessments of frailty, HFRS uses widely available objective *ICD-10 *codes, which bypasses interoperator variability.^23^ The use of *ICD-10* diagnostic codes to assess frailty also has limitations, because the HFRS was designed excluding patients younger than 75 years or with mental health diagnoses, and *ICD-10* codes generally do not capture functional ability or disease severity or polypharmacy, which are important domains of frailty assessments. Finally, the HFRS is dependent on *ICD-10* codes entered before the hospitalization, rendering it unavailable for individuals without recent hospitalizations.^23^ A benefit to using the CFS is that it is based on clinical data obtained directly from the individual or caregiver (self-reported functional status), and it is easy to navigate.^22^ However, it requires dedicated clinical data collection, can be affected by interoperator variability, and requires that the user be educated on and understand the frailty paradigm.^2^

The Canadian Frailty Network states, “To address frailty, we must recognize when it occurs, increase awareness of its significance, develop holistic models of care, and generate better evidence for its treatment.”^6^ The first step is to accurately identify frailty and select the most appropriate clinical tool for measurement. On the basis of our results, the HFRS administrative data–based frailty assessment tool cannot fully replace the bedside assessment of frailty by CFS, specifically in the cohort of patients deemed vulnerable, for whom interventions such as pulmonary rehabilitation may be important. The HFRS is more specific at detecting frailty (using a score >5 points) and can be easily calculated on a population level with administrative data. However, the poor sensitivity calls into question whether the HFRS would provide value as a widespread screening tool for frailty in the population of hospitalized patients with COPD exacerbation. This finding is corroborated by a recent study^27^ that found poor agreement between the HFRS and CFS for patients in the intensive care unit. In addition, the HFRS was less able to estimate 1-year survival, which suggests that clinical bedside frailty assessment may still be necessary for accurate assessments in some clinical settings.^27^ Further work is needed to determine whether this observation applies to comparisons between other administrative data–based frailty screening tools and bedside assessment tools.

Among individuals deemed moderately or severely frail by the CFS measurement in our cohort, a larger proportion were found to be women (73.5%). To our knowledge there are no previous studies that have described sex differences in frailty among people with COPD or the implications on quality of life, morbidity, and mortality. The literature suggests that women in the general population have higher frailty scores than men when matched for age, despite lower mortality rates.^28,29^ This sex difference has also been reported with regard to frailty among patients with heart failure.^30^ Further investigation into degrees of frailty between sexes and the implications of those differences among those with COPD is warranted to inform models of care.

### Strengths and Limitations

A strength of our study is that it compares a population-based administrative data screening tool with a bedside assessment tool in a clinical practice patient cohort, whereas the majority of studies use a single frailty assessment tool. We also used methods to attempt to improve the HFRS scoring system with an optimal probability threshold.

Our study also has several limitations. First, we conducted our analysis among hospitalized patients who used a COPD clinical pathway during hospitalization, which represented 48.3% of the hospitalizations for COPD exacerbation during the study time frame. This may have introduced selection biases that could not be accounted for in this observational study. We attempted to evaluate this by summarizing the mean age, sex proportion, and median Elixhauser Comorbidity Index scores in the patients who were not included in the study (Figure 1). The aggregate demographic profile is similar (female proportion, 56.6% vs 53.8%; mean age range, 66.9-72.7 years vs 71.8 years, in the included vs excluded cohorts); however, the possibility of bias still remains. Next, the HFRS is a validated tool in a population older than 75 years, whereas the majority of patients in our cohort were younger than that threshold. This may have contributed to suboptimal detection of frailty by the HFRS. Because frailty is not solely based on age and can occur in younger populations, especially in those with chronic disease, this highlights the need for validation of frailty assessment tools in younger individuals. Next, we studied a hospitalized cohort of patients and used the CFS to determine frailty on the basis of functional status 2 weeks before hospitalization. Given that patients were acutely unwell, it is possible that their recollection of functional status at baseline was influenced by their current illness state, introducing a recall bias toward higher degrees of frailty. Furthermore, the CFS was determined from the medical record by consensus of 2 physicians, and we did not determine agreement in scores between the 2 independent assessors to overcome any potential subjectivity in ratings. In addition, our sample size was small and the applicability of the findings, including the range of frailty detected, may be limited by the fact that we included patients from only a single tertiary center. Multicenter, large cohort studies comparing multiple administrative and clinical frailty tools are warranted among individuals with COPD to determine the optimal methods to detect frailty.

## Conclusions

In this cross-sectional study, among hospitalized patients with COPD exacerbation, the administrative data–based HFRS instrument demonstrated poor sensitivity in detecting frailty as measured by the functional status–based bedside CFS instrument. Using the HFRS to detect frailty among hospitalized patients with COPD exacerbation may result in missed opportunities to provide interventions, such as pulmonary rehabilitation and care planning earlier in the chronic disease trajectory, which can potentially improve the quality of life for patients with COPD. To improve bedside frailty recognition and detection among individuals with COPD, research should focus on adapting existing validated bedside frailty assessment tools to optimize detection of frailty in younger populations, explore the performance of these tools in male vs female patients, and study whether early detection of frailty combined with optimal interventions can subsequently improve clinical outcomes in the chronic disease trajectory.

## Notes

Supplement 1. **eFigure.** The Clinical Frailty Scale by Rockwood et al **eTable.** Cross-Tabulation Between the HFRS and CFS to Calculate the Sensitivity and Specificity of the HRFS in Detecting ‘Frail’ and ‘Non Frail’ Individuals Based on CFS Scoring

Supplement 2.
Data Sharing Statement
