# Supplementary material for: Comparing the Hospital Frailty Risk Score and the Clinical Frailty Scale Among Older Adults With Chronic Obstructive Pulmonary Disease Exacerbation
Source: JAMA Netw Open. 2023 Feb 2;6(2):e2253692. doi: 10.1001/jamanetworkopen.2022.53692 (PMC9896302; doi:10.1001/jamanetworkopen.2022.53692)
Supplement: Supplement 1. — eFigure. The Clinical Frailty Scale by Rockwood et al eTable. Cross-Tabulation Between the HFRS and CFS to Calculate the Sensitivity and Specificity of the HRFS in Detecting ‘Frail’ and ‘Non Frail’ Individuals Based on CFS Scoring [file jamanetwopen-e2253692-s001.pdf]

Supplemental Online Content

Chin M, Kendzerska T, Inoue J, et al. Comparing the hospital frailty risk score and the clinical frailty scale among older adults with chronic obstructive pulmonary disease exacerbation. *JAMA Netw Open*. 2023;6(2):e2253692. doi:10.1001/jamanetworkopen.2022.53692

eFigure. The Clinical Frailty Scale by Rockwood et al

eTable. Cross-Tabulation Between the HFRS and CFS to Calculate the Sensitivity and Specificity of the HRFS in Detecting 'Frail' and 'Non Frail' Individuals Based on CFS Scoring

This supplemental material has been provided by the authors to give readers additional information about their work.

© 2023 Chin M et al. *JAMA Network Open.*

eFigure. The Clinical Frailty Scale by Rockwood et al

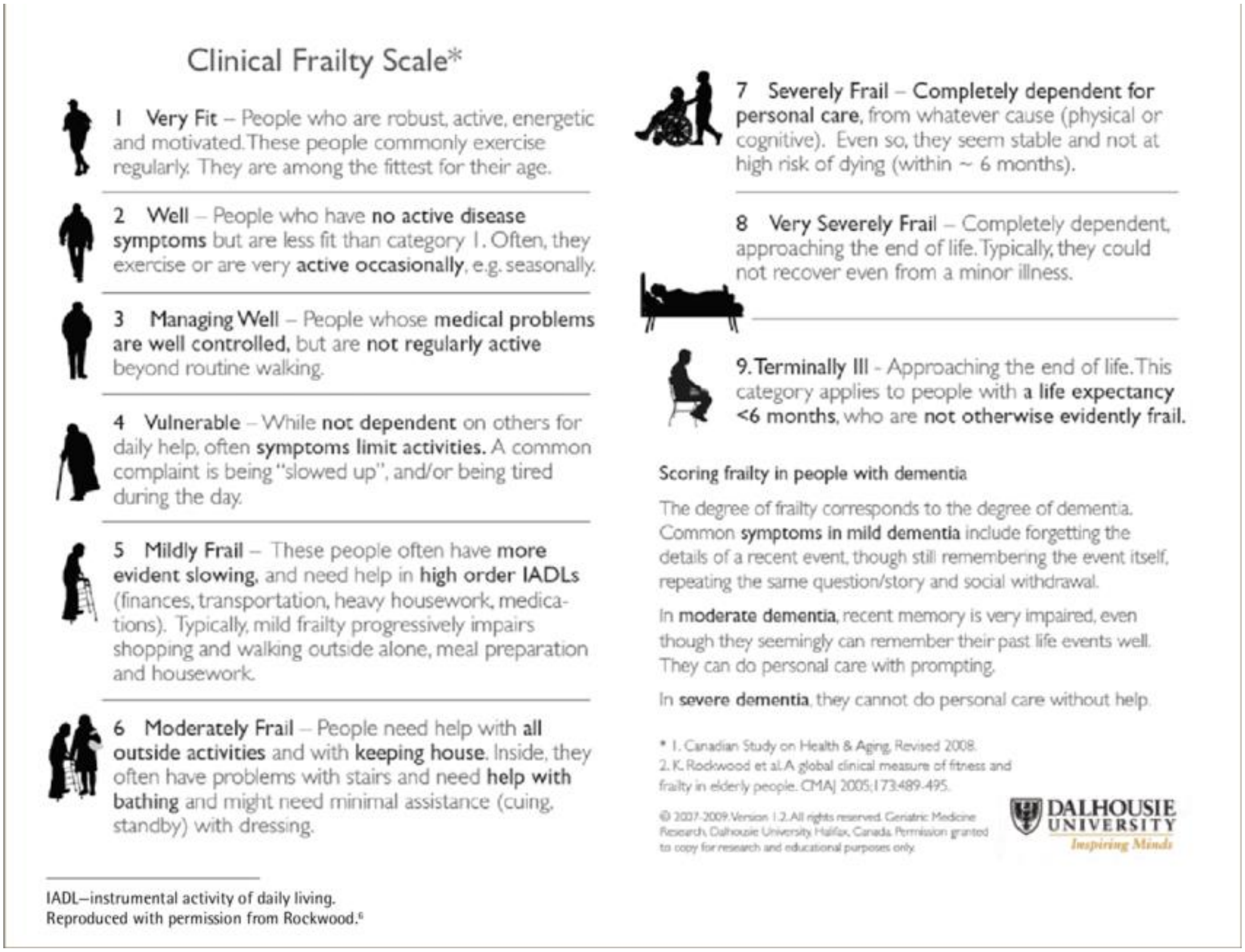

Clinical Frailty Scale*

1 **Very Fit** – People who are robust, active, energetic and motivated. These people commonly exercise regularly. They are among the fittest for their age.

2 **Well** – People who have **no active disease symptoms** but are less fit than category 1. Often, they exercise or are very **active occasionally**, e.g. seasonally.

3 **Managing Well** – People whose **medical problems are well controlled**, but are **not regularly active** beyond routine walking.

4 **Vulnerable** – While **not dependent** on others for daily help, often **symptoms limit activities.** A common complaint is being "slowed up", and/or being tired during the day.

5 **Mildly Frail** – These people often have **more evident slowing**, and need help in **high order IADLs** (finances, transportation, heavy housework, medications). Typically, mild frailty progressively impairs shopping and walking outside alone, meal preparation and housework.

6 **Moderately Frail** – People need help with **all outside activities** and with **keeping house**. Inside, they often have problems with stairs and need **help with bathing** and might need minimal assistance (cuing, standby) with dressing.

7 **Severely Frail** – **Completely dependent for personal care**, from whatever cause (physical or cognitive). Even so, they seem stable and not at high risk of dying (within ~ 6 months).

8 **Very Severely Frail** – Completely dependent, approaching the end of life. Typically, they could not recover even from a minor illness.

9. **Terminally Ill** - Approaching the end of life. This category applies to people with **a life expectancy <6 months**, who are **not otherwise evidently frail.**

Scoring frailty in people with dementia

The degree of frailty corresponds to the degree of dementia. Common **symptoms in mild dementia** include forgetting the details of a recent event, though still remembering the event itself, repeating the same question/story and social withdrawal.

In **moderate dementia**, recent memory is very impaired, even though they seemingly can remember their past life events well. They can do personal care with prompting.

In **severe dementia**, they cannot do personal care without help.

* 1. Canadian Study on Health & Aging, Revised 2008.
2. K. Rockwood et al. A global clinical measure of fitness and frailty in elderly people. CMAJ 2005;173:489-495.

© 2007-2009. Version 1.2. All rights reserved. Geriatric Medicine Research, Dalhousie University, Halifax, Canada. Permission granted to copy for research and educational purposes only.

DALHOUSIE UNIVERSITY
Inspiring Minds

IADL—instrumental activity of daily living.
Reproduced with permission from Rockwood.[6]

eTable. Cross-Tabulation Between the HFRS and CFS to Calculate the Sensitivity and Specificity of the HRFS in Detecting 'Frail' and 'Non Frail' Individuals Based on CFS Scoring

	CFS<br>Vulnerable/Mild/Mod/Severe Frailty	CFS<br>Managing Well/Well
HFRS Frail<br>(Intermediate and High Risk)	**23**	**1**
HFRS Not Frail<br>(Low Risk)	**62**	**13**
	**Sensitivity**	**Specificity**
	**27.1%**	**92.9%**

© 2023 Chin M et al. *JAMA Network Open.*
