# Supplementary material for: Comparing the Hospital Frailty Risk Score and the Clinical Frailty Scale Among Older Adults With Chronic Obstructive Pulmonary Disease Exacerbation
Source: JAMA Netw Open. 2023 Feb 2;6(2):e2253692. doi: 10.1001/jamanetworkopen.2022.53692 (PMC9896302; doi:10.1001/jamanetworkopen.2022.53692)
Supplement: Supplement 2. — Data Sharing Statement [file jamanetwopen-e2253692-s002.pdf]

Data Sharing Statement

Chin. Comparing the Hospital Frailty Risk Score and the Clinical Frailty Scale Among Older Adults With Chronic Obstructive Pulmonary Disease Exacerbation. *JAMA Netw Open*. Published February 02, 2023. doi:10.1001/jamanetworkopen.2022.53692

Data
Data available: No
